# CCDC50, an essential driver involved in tumorigenesis, is a potential severity marker of diffuse large B cell lymphoma

**DOI:** 10.1007/s00277-023-05409-w

**Published:** 2023-09-09

**Authors:** Yuqi Gong, Hongyan Tong, Fang Yu, Qi Liu, Xianbo Huang, Guoping Ren, Zhongqin Fan, Zhe Wang, Jing Zhao, Zhengrong Mao, Jing Zhang, Ren Zhou

**Affiliations:** 1 Department of Pathology, The First Affiliated Hospital, Zhejiang University School of Medicine, Hangzhou, China; 2 Department of Pathology and Pathophysiology, Zhejiang University School of Medicine, Hangzhou, China; 3 Department of Hematology, The First Affiliated Hospital, Zhejiang University School of Medicine, Hangzhou, China; 4 Department of Pathology, Xijing Hospital, Fourth Military Medical University, Xi’an, China; 5 Department of Pathology, The Children’s Hospital, Zhejiang University School of Medicine, Hangzhou, China

**Keywords:** DLBCL, Biomarker, CCDC50, c-Myc, Exosome

## Abstract

Diffuse Large B Cell Lymphoma (DLBCL) is the most common form of blood cancer. Among the subtypes, the activated B-cell (ABC) subtype is typically more aggressive and associated with worse outcomes. However, the underlying mechanisms are not fully understood. In this study, we performed microarray analysis to identify potential ABC-DLBCL-associated genes. We employed Kaplan–Meier methods and cox univariate analysis to explore the prognostic value of the identified candidate gene Coiled-coil domain containing 50 (CCDC50). Additionally, we used DLBCL cell lines and mouse models to explore the functions and mechanisms of CCDC50. Finally, we isolated CCDC50-bearing exosomes from clinical patients to study the correlation between these exosomes and disease severity. Our results demonstrated that CCDC50 not only showed significantly positive correlations with ABC subtype, tumor stage and number of extranodal sites, but also suggested poor outcomes in DLBCL patients. We further found that CCDC50 promoted ABC-DLBCL proliferation in vitro and in vivo. Mechanistically, CCDC50 inhibited ubiquitination-mediated c-Myc degradation by stimulating the PI3K/AKT/GSK-3β pathway. Moreover, CCDC50 expression was positively correlated with c-Myc at protein levels in DLBCL patients. Additionally, in two clinical cohorts, the plasma CCDC50-positive exosomes differentiated DLBCL subtypes robustly (AUC > 0.80) and predicted disease severity effectively (*p* < 0.05). Our findings suggest that CCDC50 likely drives disease progression in ABC-DLBCL patients, and the CCDC50-bearing exosome holds great potential as a non-invasive biomarker for subtype diagnosis and prognosis prediction of DLBCL patients.

**Supplementary Information:**

The online version contains supplementary material available at 10.1007/s00277-023-05409-w.

## Introduction

Diffuse Large B Cell Lymphoma (DLBCL), the most common type of non-Hodgkin lymphoma (NHL), account for 30 ~ 40% of all newly diagnosed cases worldwide [1]. Despite the standard frontline chemoimmunotherapy regimen (R-CHOP) improving the outcomes of DLBCL patients, up to 40% of patients still succumb to this disease. Immunohistochemistry analysis categorizes DLBCL into GCB and non-GCB subtypes based on the expression levels of MUM1, BCL6, and CD10 [2]. Non-GCB subtype patients have a shorter survival time. Gene expression profiling has identified two major subtypes: germinal center B cell-like (GCB) and activated B cell-like (ABC) DLBCL [3]. ABC-DLBCL is more chemo-resistant and has an inferior prognosis compared to the GCB subtype, with a 3-year progression-free survival rate of 40% versus 75% (*p* < 0.001) [1, 4–6]. Despite significant advances over the past few decades, oncogenic aberrations in ABC-DLBCL progression are not fully characterized. Thus, the ongoing effort is required to identify new biomarkers associated with this aggressive subtype.

Currently, the diagnosis of DLBCL subtypes primarily relies on invasive procedures, such as fine needle aspiration, core biopsy, and lymph node excision. These methods present challenges in obtaining a quick and accurate diagnosis. Thus, the identification of sensitive biomarkers with non-invasive ways, such as reliable liquid biopsy, is highly desired. Numerous studies have shown that tumor derived exosomes can be effective biomarkers for cancer diagnosis [7]. However, their potential to diagnose and predict the progression of DLBCL remains poorly defined.

CCDC50 is a tyrosine-phosphorylated protein with multiple ubiquitin-interacting domains [8]. Recent studies have shown that CCDC50 acts as a new autophagy receptor, suppressing antiviral signaling by negatively regulating RIG-I/MADA5 [9], cGAS-STING [10], and NLRP3 [11]. In Hepatocellular Carcinoma (HCC) [12], Mantle Cell Lymphoma (MCL) and Chronic Lymphocytic Leukemia (CLL) [13], CCDC50 is required for cell survival. However, its function and molecular consequences in DLBCL remain unclear to date.

In this study, we present the following discoveries: (1) CCDC50 is increased in ABC-DLBCL and associated with poor prognosis, (2) CCDC50 stabilizes c-Myc protein in a PI3K/AKT/GSK-3β dependent manner, leading to the proliferation of ABC-DLBCL, and (3) DLBCL-derived CCDC50-positive exosomes in plasma effectively distinguish DLBCL subtypes and predictes patient severity. These findings may help develop a new therapeutic strategy to treat ABC-DLBCL and provide a non-invasive approach for subtype diagnosis and prognostic monitoring of DLBCL.

## Materials and methods

### Data collection and bioinformatic analysis

GSE10846, GSE19246, GSE32918, GSE50721, GSE64820, and GSE94669, were downloaded from the Gene Expression Omnibus database (https://www.ncbi.nlm.nih.gov/geo/). DLBCL datasets from the GEPIA (Gene Expression Profiling Interactive Analysis) (http://gepia.cancer-pku.cn) and the TCGA (The Cancer Genome Atlas) (https://xenabrowser.net/datapages/) were also used in this study. A total of 284 paraffin-embedded tissue samples and 129 plasma samples of DLBCL patients were collected from The First Affiliated Hospital, Zhejiang University School of Medicine.

Limma was used to identify differentially expressed genes, and two R packages (survival, survminer) were used to plot Kaplan–Meier curves. GSE10846 and 65 paraffin-embedded tissue samples with cilinical information were used for survival analysis.

Patients were divided into the high and low expressed groups according to the transcriptional value of CCDC50 (high: value > upper quartiles, low: value < lower quartiles). GSEA software (version 4.0.3) was used to identify tumor-related pathways in the CCDC50 high and low groups.

### Cell culture, plasmids, transfection

Human DLBCL cell lines (SU-DHL-2, OCI-LY3, OCI-LY10, SU-DHL-4, Pfeiffer, and Toledo) were grown in IMDM with 10% FBS. HEK293T and Hela cells were grown in DMEM with 10% FBS. SU-DHL-2, Pfeiffer, and Toledo were purchased from Shanghai EK-Bioscience Co., Ltd; SU-DHL4 was obtained from the Type Culture Collection of the Chinese Academy of Sciences; OCI-Ly3 and OCI-LY10 were kindly provided by Dr. Zhe Wang from the Department of Pathology at Xijing Hospital, the Fourth Military Medical University. HEK293T and Hela were kindly provided by Dr. Chengfang Dong from Department of Pathology, Zhejiang University School of Medicine. All the cells were grown in a humidified atmosphere with 5% CO2 at 37 °C.

The shRNA sequence was cloned into pLVX-shRNA1. The sequence of c-Myc (NM_ 001354870.1) and CCDC50 (NM_174908.4) were cloned into Plvx-IRES-Neo and pLVX-TRE3G, respectively. Stable transfection cells were selected with puromycin (2 μg/ml) and/or G418 (400 μg/ml). Lipo3000 Transfection Reagent (Invitrogen) was used for lentivirus production. Sequence information of shRNA is summarized in Table S1.

### Real-time qPCR, Western blotting (WB), CCK8, EdU assays, Immunoprecipitation, immunofluorescence (IF), Immunohistochemistry (IHC), and antibodies

Experiments were performed as described previously [14]. In our Real-time qPCR experiments, the primer sequences employed are summarized in Table S1. The primer for the c-Myc was adopted from a previously published literature [15]. The primer of CCDC50 was designed according to the sequence of CCDC50 variant 1. We then performed PrimerBLAST analysis to ensure its accurateness and specificity. The concentration of antibodies used in IF: c-Myc (1:100, ab32072). The concentration of antibodies used in IHC: CCDC50 (1:500, ab127169), c-Myc (1:100, ab32072). The Staining value (values 0–12) in IHC experiments was calculated by the product of the intensity of CCDC50 positive staining (negative, 0; weak 1; moderate, 2; strong, 3 scores) and the proportion of immunopositive cells of interest (< 25%, 1; 25–50%, 2; 50–75%, 3; > 75%, 4 scores). Patients were divided into two groups according to median value of IHC staining value. Primary and secondary antibodies used in this study were listed in Table S2.

### Xenograft studies in NOD/SCID mice

5 × 10^6^ cells were collected and mixed with 50uL PBS and 50uL Matrigel (Corning 354234). Cells transfected with shNTC were injected into the right flank of 6–8 weeks old NOD-SCID mice, and cells transfected with shCCDC50 were injected into the left flank. The tumor volumes were measured every 5 days after 5 days post engraftment.

### Inhibitor and ubiquitination assay

Cycloheximide (CHX, 100ug/ml, MCE) was used to inhibit protein synthesis. MG132 (10 μM, MCE) was used to inhibit proteasome proteolytic activity. CHIR-99021 (1 μM, MCE) was used to inhibit GSK-3β activity.

1 × 10^6^ cells were seeded per well in 6-well plates and transfected with indicated plasmids using PolyJetTM DNA In Vitro Transfection Reagent (SigaGen). After a 48-h incubation period, 10uM of MG132 was added to the culture medium, and cells were incubated for an additional 6 h. Then, cells were lysed in cell lysis buffer (Beyotime, P0013) and subjected to immunoprecipitation using an anti-c-Myc antibody (Abcam, ab32072). The anti-K48-Ub antibody (CST #4289) was used to detect the ubiquitination levels of c-Myc.

### Exosome extraction, nanoparticle tracking analysis, and Apogee nanoscale flow cytometry analysis

The protocols of these experiments were detailed in our recent study [16]. We also summarized the exosome extraction and analysis in Supplementary file 1. Specifically, rabbit anti-CCDC50 monoclonal antibodies were labeled with Zenon Alexa Fluor 647. Rabbit anti-IgG monoclonal antibodies were also labeled as negative controls.

### Electron microscopy

10 μL of exosome liquid was deposited onto a 300-mesh carbon-coated copper grid and incubated for 1 min. Subsequently, the exosome liquid was carefully removed using filter papers. Then, 10 μL of a 2% uranyl acetate staining solution was deposited to the copper grid and incubated for 1 min, followed by removal of the solution using filter paper. Next, 10 μL of staining solution was deposited to the copper grid and incubated for 1 min, after which the staining solution was removed using filter paper. Finally, the morphology of the exosomes was observed using a transmission electron microscope (Talos 120 kV cryo-TEM).

### Statistical analysis

All statistical analyses including unpaired t-test, one-way ANOVA, Pearson and Spearman correlation analyses, and Chi-Square Test were performed using GraphPad Prism9 software (version 9.2.0). Experiments were repeated independently no less than three times with results presented as the mean ± SD. *P* < 0.05 was considered statistically significant. The following symbols were used to denote the level of significance: *p < 0.05, **p < 0.01, ***p < 0.001, ****p < 0.0001.

## Results

### CCDC50 is upregulated in ABC-DLBCL and associated with inferior prognosis

**Fig. 1:**
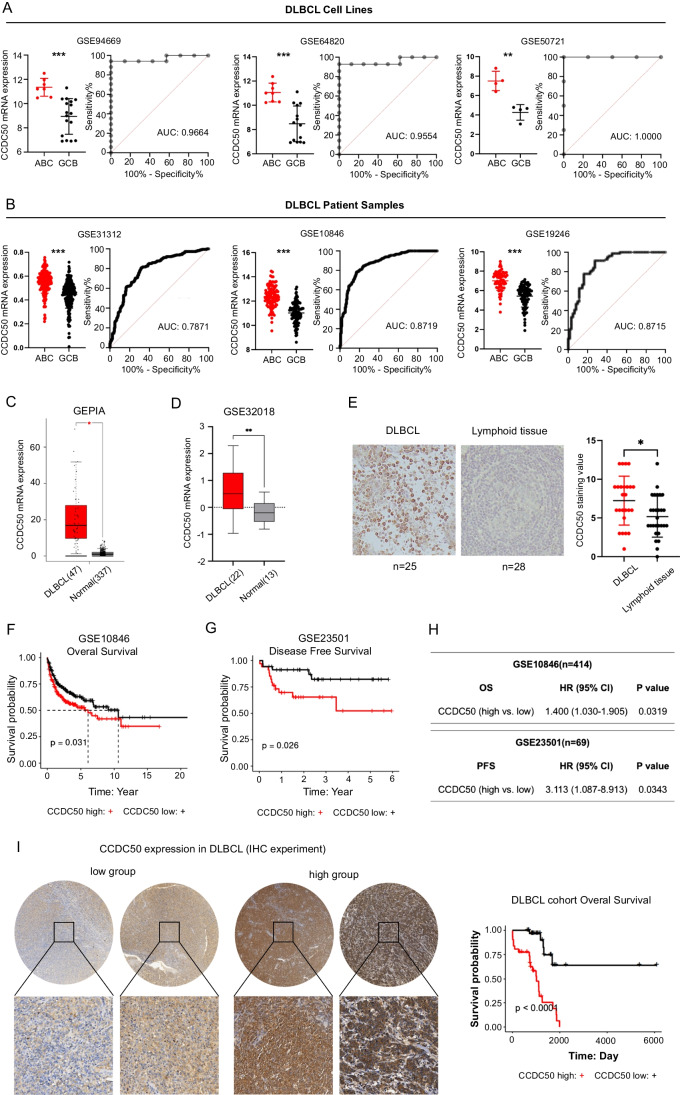
CCDC50 is upregulated in ABC-DLBCL and associated with poor prognosis. Dot plots indicated CCDC50 mRNA expression in DLBCL subtypes and ROC curves indicated the accuracy of DLBCL subtype diagnosis in cell line datasets (**A**), and patient datasets (**B**). CCDC50 mRNA (**C**, **D**) and protein levels (**E**) in DLBCL and normal samples. Kaplan–Meier survival analysis (**F**, **G**) and cox univariate analysis (**H**) of CCDC50 mRNA expression in DLBCL datasets. (**I**) Kaplan–Meier survival analysis of CCDC50 protein expression in DLBCL patients

We conducted bioinformatic and survival analyses to identify driver oncogenes associated with ABC-DLBCL (Fig. S1). In this study, we found that CCDC50 effectively discriminate ABC from GCB-DLBCL with AUC values ranging from 0.7871 to 1.0000 (Fig. 1A-B). Analyses of two independent datasets showed that CCDC50 mRNA levels were significantly elevated in DLBCL patients compared to healthy controls (Fig. 1C-D). Similarly, CCDC50 protein levels were higher in DLBCL than in lymphoid tissues (Fig. 1E). Next, we investigated the relationships between CCDC50 and clinicopathologic features in patients (GSE10846, Table S3). We observed significant correlations between CCDC50 expression and DLBCL subtype, tumor stage and number of extranodal sites (*p* < 0.05, Table 1, *n* = 414). Kaplan–Meier survival analysis revealed that patients with higher CCDC50 expression showed shorter overall survival (*p* = 0.031, Fig. 1F, Supplementary file 2) and disease free survival time (*p* = 0.026, Fig. 1G). Furthermore, the results of cox univariate analysis corroborated these findings, clearly identifying CCDC50 as a significantly risk factor for DLBCL patients (Fig. 1H). Our IHC experiment also showed that higher CCDC50 expression is significantly associated with shorter overall survival time in DLBCL (*p* < 0.0001, Fig. 1I, Supplementary file 3). Collectively, our results suggest that CCDC50 is significantly elevated in DLBCL, especially in ABC-DLBCL, and is a predictor of worse prognosis.

**Table 1:** Relationships between the expression level of CCDC50 and clinicopathologic features

Clinical feature	Expression of CCDC50				*P*-value
	Low expression		High expression		
	Number	Percentage	Number	Percentage	
Gender					
Male	104	26.26%	120	30.30%	
Female	89	22.47%	83	20.96%	0.2942
Age(years)					
≤ 60	98	23.67%	90	21.74%	
> 60	109	26.33%	117	28.26%	0.4297
Subtype					
GCB	143	40.86%	40	11.43%	
ABC	28	8.00%	139	39.71%	** < 0.0001**
ECOG performance status					
0–2	183	47.04%	173	44.47%	
3–4	12	3.08%	21	5.40%	0.0983
Stage					
1–2	109	26.85%	79	19.46%	
3–4	94	23.15%	124	30.54%	**0.0028**
LDH ratio					
≤ median ration(1.01)	96	27.35%	78	22.22%	
> median ration(1.01)	82	23.36%	95	27.07%	0.0975
Number of extranodal sites					
< 1	136	35.51%	102	26.63%	
≥ 1	55	14.36%	90	23.50%	**0.0003**

*GCB:* Germinal center B cell-like; *ABC:* Activated B cell-like; *ECOG:* Eastern Cooperative Oncology Group; The *P*-value < 0.05 was highlighted in bold font to underscore its significance

### CCDC50 promotes ABC-DLBCL proliferation in vitro and in vivo

**Fig. 2:**
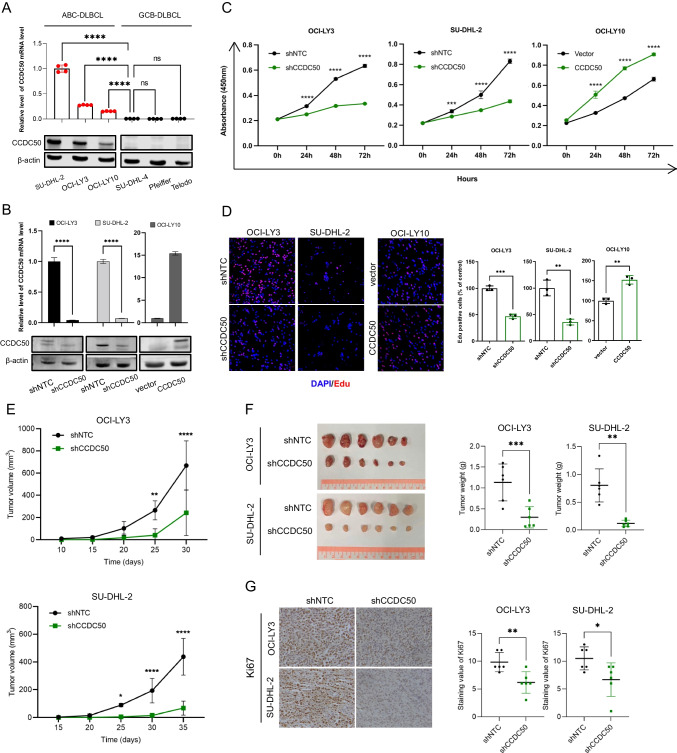
CCDC50 promotes ABC DLBCL proliferation in vitro and in vivo. (**A**) CCDC50 expression in DLBCL cell lines. (**B**) CCDC50 mRNA and protein levels in shNTC-/shCCDC50-OCI-LY3 cells, shNTC-/shCCDC50-SU-DHL-2 cells, and vector/CCDC50-OCI-LY10 cells. CCK8 (**C**) and EdU assays (**D**) were performed to detect the proliferation of shNTC-/shCCDC50-OCI-LY3 cells, shNTC-/shCCDC50-SU-DHL-2 cells, and vector/CCDC50-OCI-LY10 cells. (**E**) The tumor growth curve. (**F**) tumor weight. (**G**) Tumor tissue Ki67 IHC staining in NOD-SCID mice

We performed RT-qPCR and WB analysis to assess CCDC50 expression in DLBCL cell lines. Results revealed that both CCDC50 mRNA and protein levels were significantly higher in ABC-DLBCL cell lines (Fig. 2A). We then selected ABC-DLBCL cell lines for further study. We knocked down CCDC50 in OCI-LY3/SU-DHL-2 and overexpressed it in OCI-LY10 (Fig. 2B). Results of CCK8 and EdU assays showed that CCDC50 knockdown significantly reduced cell proliferation, while CCDC50 overexpression remarkably increased it (Fig. 2C, Supplementary file 4, Fig. 2D). Furthermore, we established a xenograft model by subcutaneously injecting CCDC50 stably knocked down OCI-LY3/SU-DHL-2 cells. Tumors in shCCDC50 group displayed slower growth than those in shNTC group (Fig. 2E), ultimately reducing the tumor mass (Fig. 2F). Additionally, tumors in the shCCDC50 group exhibited decreased Ki67 staining values (Fig. 2G). These results confirm that CCDC50 is essential for ABC-DLBCL proliferation both in vitro and in vivo.

### c-Myc is the downstream effector of CCDC50-induced ABC-DLBCL proliferation

**Fig. 3:**
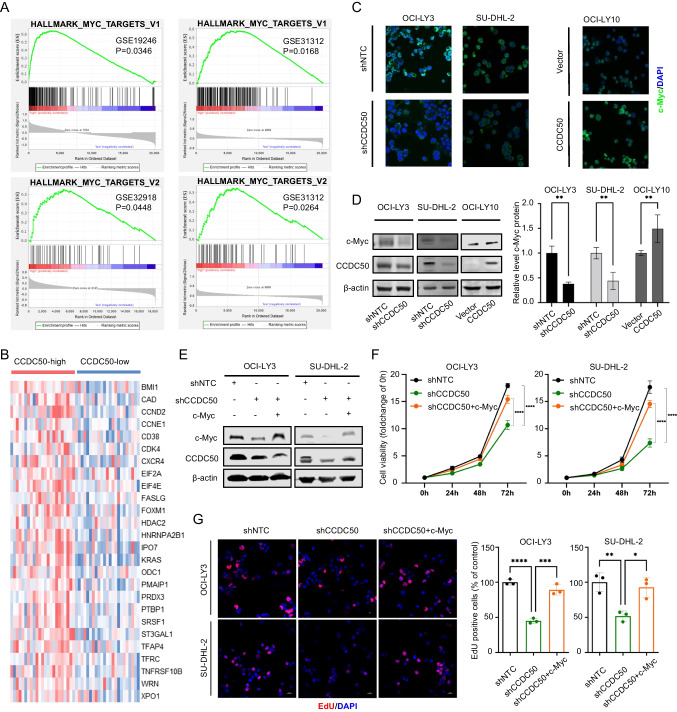
CCDC50 promotes ABC-DLBCL proliferation through positively regulate c-Myc. (**A**) Enriched pathways were detected by the GSEA approach based on patient datasets. (**B**) The heat map represented different expressions of c-Myc target genes in CCDC50-high and CCDC50-low groups in TCGA dataset. (**C**, **D**) c-Myc expression in shNTC-/shCCDC50-OCI-LY3, shNTC-/shCCDC50-SU-DHL-2, and vector/CCDC50-OCI-LY10 cells. (**E**) The stable clone with c-Myc expression was established in CCDC50-depleted cells. (**F**, **G**) The cell viability states and percentage of proliferated cells before and after overexpression of c-Myc in CCDC50-depleted cells

We performed GSEA analysis to uncover molecular mechanisms behind CCDC50-induced proliferation. Results revealed that patients with higher expression of CCDC50 exhibited significant enrichment of MYC-related pathways (Fig. 3A). Moreover, patients in the TCGA dataset with higher expression of CCDC50 showed elevated levels of c-Myc-induced genes (Fig. 3B). We hypothesized that CCDC50 promotes ABC-DLBCL proliferation via c-Myc. Immunofluorescence experiments indicated that after the knockdown of CCDC50, the levels of c-Myc decreased, whereas overexpression of CCDC50 resulted in a notable accumulation of c-Myc (Fig. 3C), which was supported by WB analysis (Fig. 3D). To confirm that c-Myc is the downstream target of CCDC50-induced ABC-DLBCL proliferation, we enforced the expression of c-Myc in cells with CCDC50 knockdown (Fig. 3E). Results of CCK8 and EdU assays showed that c-Myc recovery rescued cell proliferation arrest caused by CCDC50 knockdown (Fig. 3F-G). These findings suggest that CCDC50 promotes tumor proliferation by positively regulating c-Myc expression in ABC-DLBCL.

### CCDC50 stabilizes c-Myc protein by reducing its ubiquitination

**Fig. 4:**
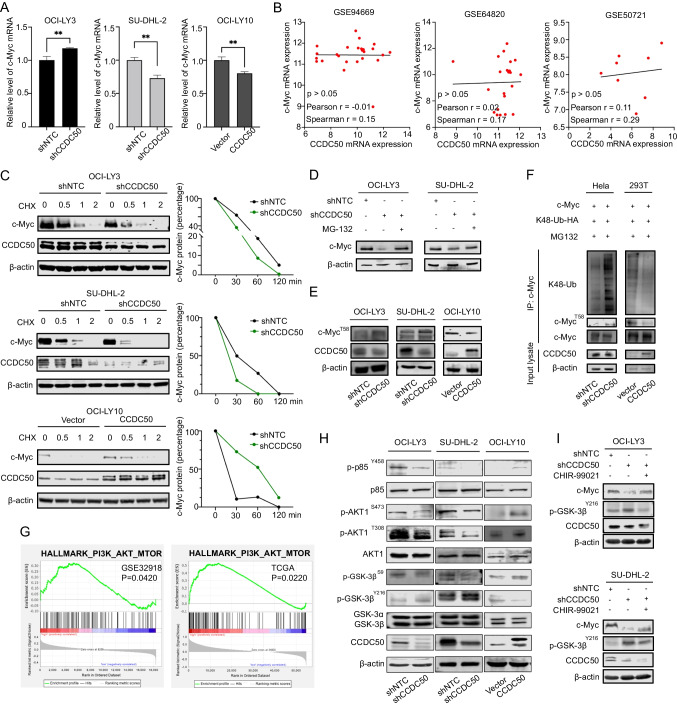
CCDC50 stabilizes c-Myc protein by reducing its ubiquitination through PI3K/AKT/GSK-3β. (**A**) c-Myc mRNA level in cells with CCDC50 knockdown or CCDC50 expression. (**B**) Correlation between c-Myc and CCDC50 at mRNA levels in DLBCL. (**C**) Cells were treated with CHX for indicated time intervals and the changes of c-Myc expression. (**D**) Cells with CCDC50 knockdown were treated with MG-132 for 4 h and the changes of c-Myc expression were examined. (**E**) The phosphorylated level of c-Myc^T58^ in cells with CCDC50 knockdown or overexpression. (**F**) Ubiquitination and phosphorylation levels of c-Myc in shNTC-/shCCDC50-Hela cells and vector-/CCDC50-HEK-293 T cells. (**G**) Enriched pathways in CCDC50-high groups in DLBCL patient. (**H**) The protein level of PI3K/AKT/GSK-3β pathways, and CCDC50 in shNTC-/shCCDC50-OCI-LY3 cells, shNTC-/shCCDC50-SU-DHL-2 cells, and vector/CCDC50-OCI-LY10 cells. (**I**) Cells with CCDC50 knockdown were treated with CHIR-99021 (1 μM) for 4 h, and the protein level of c-Myc, p-GSK-3β^Y216^ were examined

We next explore whether CCDC50 affects c-Myc expression at the transcriptional or post-transcriptional level. We found that knockdown of CCDC50 increased c-Myc mRNA expression in OCI-LY3, while overexpression of CCDC50 decreased c-Myc mRNA expression in OCI-LY10 (Fig. 4A). Meanwhile, no significant correlations were detected between CCDC50 and c-Myc mRNA (Fig. 4B), indicating that CCDC50 primarily regulates c-Myc post-translationally. Through CHX chase experiments, we observed that CCDC50 knockdown decreased the half-life of the endogenous c-Myc protein, while CCDC50 overexpression increased it (Fig. 4C). Furthermore, treatment with MG132 restored c-Myc protein levels in CCDC50-knocked down cells (Fig. 4D), indicating that the regulation may be involved in ubiquitination process. We then examined the phosphorylation of c-Myc at the threonine 58, which results in ubiquitination-mediated degradation of c-Myc. Results showed that CCDC50 knockdown increased the p-T58 level, while CCDC50 overexpression decreased it (Fig. 4E). Moreover, CCDC50 knockdown significantly increased c-Myc ubiquitination, while CCDC50 overexpression dramatically repressed it (Fig. 4F). These findings support that CCDC50 stabilizes c-Myc by preventing its ubiquitination.

GSEA analysis revealed that high CCDC50 expression is associated with activated PI3K/AKT/mTOR pathway (Fig. 4G). Since active AKT inhibits the GSK-3β [17], preventing it from phosphorylating c-Myc^T58^. CCDC50 may stabilize c-Myc protein via PI3K/AKT/GSK-3β pathway. As shown in Fig. 4H, CCDC50 knockdown reduced phosphorylation levels of p85 and AKT, while CCDC50 overexpression increased them. The level of active form p-GSK-3β^Y216^ was increased after CCDC50 knockdown, but notably decreased after CCDC50 overexpression. The change of inactive form p-GSK-3β^S9^ showed the opposite trend. Additionally, treatment with CHIR-9902 significantly restored the level of c-Myc protein in cells with CCDC50 knockdown (Fig. 4I). These findings support that CCDC50 activates PI3K/AKT/GSK-3β axis to reduce ubiquitination-mediated c-Myc degradation.

### CCDC50 expression is positively correlated with c-Myc in DLBCL

**Fig. 5:**
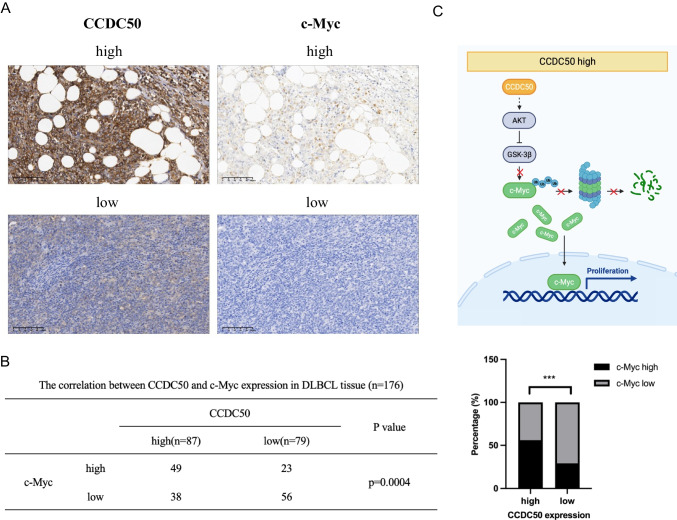
CCDC50 expression is positively correlated with c-Myc. (**A**) c-Myc expression was higher in patients with higher expression of CCDC50. (**B**) Correlation between CCDC50 and c-Myc expression were assessed with Chi-Square Test. (**C**) A proposed model to illustrate that higher expression of CCDC50 activates AKT, which inhibits GSK-3β mediated phosphorylation of c-Myc^T58^, therefore promoting c-Myc accumulation

We then performed IHC staining to examine the correlation between CCDC50 and c-Myc. Patients with high CCDC50 expression exhibit high c-Myc expression (Fig. 5A). Approximately 56.32% of patients with higher CCDC50 expression display high c-Myc expression, whereas 43.68% showed weak c-Myc staining. Similarly, 70.87% of patients with low CCDC50 expression display weak c-Myc expression, whereas 29.13% showed high c-Myc staining (Fig. 5B). Above results indicated that the protein level of CCDC50 and c-Myc were positively correlated in DLBCL patients. Thus, CCDC50 could promote DLBCL proliferation in a PI3K/AKT/GSK-3β/c-Myc dependent manner (Fig. 5C).

### Exosomal CCDC50 serves as a non-invasive diagnostic and prognostic biomarker in DLBCL

**Fig. 6:**
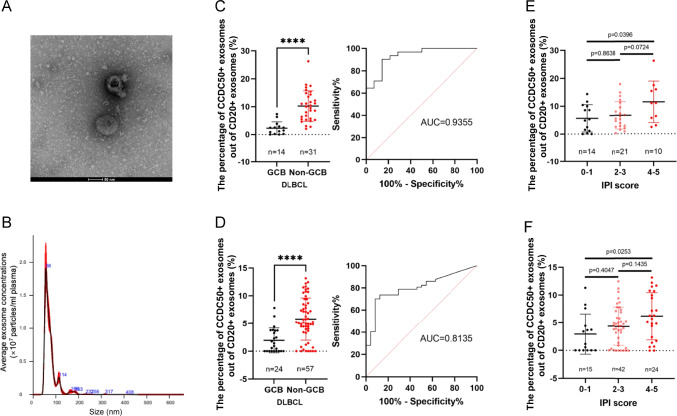
Plasma Exosomal CCDC50 protein serves as a non-invasive biomarker in DLBCL. The shape (**A**) and the diameter distribution (**B**) of plasma exosomes in DLBCL. The percentage of CCDC50 + exosomes out of CD20 + exosomes and ROC curves in the discovery (**C**) and validation cohort (**D**). The percentage of CCDC50 + exosomes out of CD20 + exosomes in different risk groups of DLBCL patients in the discovery (**E**) and validation cohort (**F**)

CCDC50 protein was found in exosomes released from DLBCL cell lines [18], prompting us to wonder whether plasma exosomes contain CCDC50 protein and whether it has clinical significance in DLBCL. We used Cryo-electron microscopy (Fig. 6A) and NTA scatter (Fig. 6B) to validate that we successfully isolated plasma exosomes from DLBCL patients. Then, based on flow cytometry analysis, we detected CCDC50 proteins in plasma exosomes (Fig. S2A). Because CD20 is a biomarker of DLBCL (Fig. S2B) and detected in plasma exosomes (Fig. S2C, Supplementary file 5), we regarded CD20 + exosomes as DLBCL-derived exosomes and CCDC50 + exosomes out of CD20 + exosomes as DLBCL-derived CCDC50 + exosomes. Results showed that the percentage of DLBCL-derived CCDC50 + exosomes were higher in Non-GCB compared to GCB in both cohorts (Fig. 6C, AUC = 0.9355; Fig. 6D, AUC = 0.8135). Moreover, based on the total IPI score, patients are divided into low- (0–1), intermediate- (2–3), and high-risk categories (4–5). We discovered that DLBCL-derived CCDC50 + exosomes were significantly higher in high-risk categories (Fig. 6E-F). Overall, the DLBCL-derived CCDC50 + exosome is a promising non-invasive biomarker with diagnostic and prognostic potential.

## Discussion

DLBCL is a complex and heterogeneous disease. The ABC-DLBCL subtype is known for its aggressive nature and poor clinical outcomes. Despite extensive research, the underlying mechanisms involved in the pathogenesis of ABC-DLBCL remain poorly understood. To explore potential biomarkers and therapeutic targets, we conducted a transcriptome analysis and identified CCDC50, a poorly investigated gene that is dysregulated in DLBCL (Fig. 1).

The human *CCDC50* gene has two isoforms: CCDC50 variant 1 (CCDC50*-*V1) contains 306 amino acids with skipping of exon 6, and CCDC50 variant 2 (CCDC50*-*V2) contains 482 amino acids. CCDC50*-*V1 is predominantly expressed and play oncogenic roles in HCC, whereas CCDC50-V2 is less expressed and has anti-tumor functions [12]. Similarly, in DLBCL, CCDC50*-*V1 is the dominant form, while CCDC50*-*V2 is rarely expressed (Fig. S3). Therefore, in this study, both knockdown and overexpression sequences of CCDC50 were targeted against CCDC50*-*V1. It has been reported that CCDC50 acts as a downstream effector of several receptors and functions as a negative regulator in NF-κB activity [19–21]. However, another study showed that CCDC50 maintains long-term activation of NF-κB signaling in Fas-stimulated thymocytes [22], and CCDC50 is required for tumor survival and activates NF-κB signaling in CLL and MCL [13]. The above data suggests that CCDC50 functions differently depending on the cellular context. In this study, we found that CCDC50 accelerates ABC-DLBCL proliferation both in vitro and in vivo (Fig. 2).

Subsequently, in exploring the biological mechanism of CCDC50 in ABC-DLBCL, we present multiple evidence supporting that CCDC50 could stabilize c-Myc to promote tumor proliferation (Fig. 3). The protein c-Myc plays critical roles in maintaining normal cell functions, including controlling cell growth and determining cell fate. The rapid degradation of c-Myc by the ubiquitin–proteasome system is essential for maintaining its normal physiological level [23, 24]. This degradation process involves sequential phosphorylation of c-Myc, starting with the stabilization of c-Myc upon the phosphorylation of S62 by EKR [25], followed by the GSK-3β mediated T58 phosphorylation [26], and finally recognition by E3 ligases [27, 28] and degradation by 26S proteasome. In many cancers, the molecules involved in c-Myc degradation pathways are dysregulated, leading to increased c-Myc stability. So far, direct targeting of c-Myc has not yet been successful due to its disordered structure and lack of specific enzymatic activity [29]. Hence, disrupting the molecules that regulate c-Myc degradation may have therapeutic benefits. In this study, we found that CCDC50 activates the PI3K/AKT pathway in DLBCL. Then, active AKT inhibits GSK3β function and reduces GSK3β-mediated phosphorylation of c-Myc^T58^ (Fig. 4). Hence, our findings provide insight into the underlying mechanism of CCDC50-induced proliferation in ABC-DLBCL and further advance the understanding of the regulatory mechanisms that govern c-Myc stability.

Given the reliability and effectiveness of CCDC50 as a biomarker in DLBCL, we aspire to conduct further investigations to determine the feasibility of its application as a clinical biomarker.

Liquid biopsy is a minimally invasive detection method that analyzes various biomarkers in biofluids for disease diagnosis and prognosis monitoring [30]. Exosomes are important source of biomarkers in liquid biopsy as they carry multiple molecules of parent cells and are enriched in biofluids [7, 31, 32]. By analyzing the contents in exosomes, researchers can identify biomarkers for diseases. In DLBCL, several studies explored the clinical value of plasma exosomes. Feng Y found that exosomal miR-99a-5p and miR-125b-5p were effective in distinguishing chemosensitive and chemoresistant DLBCL, with AUC values of 0.744 and 0.7802, respectively, and both were associated with poor outcomes [33]. Cao D identified five exosomal miRNAs (miR‐379‐5p, miR‐135a‐3p, miR‐4476, miR‐483‐3p, and miR‐451a) that were differentially expressed in DLBCL patients and healthy individuals, with AUC values greater than 0.85 [34]. Carvalho reported that exosomes from ABC-DLBCL cell lines contained more CCDC50 proteins than GCB cell lines, indicating that DLBCL-derived exosomes carry CCDC50 proteins and exosomal CCDC50 showed significantly different levels in DLBCL subtypes [18]. Therefore, we questioned whether CCDC50 is present in the exosomes in DLBCL patients? If CCDC50 is present, whether these exosomes can serve as biomarkers when they enter the bloodstream? In this study, we discovered the presence of CCDC50 + exosomes derived from DLBCL in patients plasma. Further analysis revealed that these exosomes showed higher levels in Non-GCB DLBCL and higher risk patients (Fig. 6C-F). Taken together, these findings support that the DLBCL-derived CCDC50 + exosome could be a promising non-invasive biomarker for predicting patient outcomes and distinguishing between different subtypes of DLBCL. While our consideration of CD20 + exosomes as originating from DLBCL was supported by the significantly higher expression of CD20 in DLBCL compared to corresponding normal tissues (Fig. S2B), we acknowledge the potential for CD20 expression overlap on exosomes derived from both DLBCL and normal B-cells. As such, further research efforts should aim to identify a more specific biomarker for DLBCL, ultimately enhancing diagnostic accuracy in clinical settings.

Our work provides evidence that CCDC50 contributes to the proliferation of ABC-DLBCL by reducing ubiquitination-mediated c-Myc degradation. More importantly, our analysis of more than 1000 patient samples suggests that CCDC50 may serve as a promising diagnostic and prognostic biomarker in DLBCL. Therefore, the evaluation of CCDC50 as a treatment strategy for DLBCL patients warrants further clinical investigation.

### Supplementary Information

Below is the link to the electronic supplementary material.

Fig. S1 The screening process of ABC-DLBCL-associated genes. (A) The work flow of the screening process. (B)The four genes in the left column were well investigated in ABC-DLBCL, the four genes in the middle-left column were reported in ABC-DLBCL, the nine genes in the middle-right column were mentioned in ABC-DLBCL, the thirteen genes in the right column never be reported in ABC-DLBCL. (PNG 482 kb)

High resolution image (EPS 4163 kb)

Fig. S2 Detection of Exosomal CD20 and CCDC50 proteins. (A) Levels of exosomal CD20 and CCDC50 proteins and their corresponding controls (IgG) were detected in DLBCL patient samples by flow cytometry. (B) The expression level of CD20 in 31 types of cancer. (C) The percentage of CD20+ exosomes out of all exosomes were detected in DLBCL patients and normal samples. (PNG 725 kb)

High resolution image (EPS 2871 kb)

Fig. S3 The expression level of CCDC50 V1 and V2 in DLBCL (TCGA). The expression level of each exon (A) and junction (B) of CCDC50 in DLBCL patients. (C) The expression level of CCDC50 variants and the proportion of each variant in DLBCL patients. (PNG 219 kb)

High resolution image (EPS 3015 kb)

Supplementary file1 (DOC 31 kb)

Supplementary file2 (DOC 41 kb)

Supplementary file3 (DOC 43 kb)

Supplementary file4 The process of exosome extraction and analysis. (DOCX 260 kb)

Supplementary file5 The raw data of Fig. 1F. (CSV 12 kb)

Supplementary file6 The raw data of Fig. 1I. (XLSX 10 kb)

Supplementary file7 The raw data of Fig. 2C.(XLSX 1029 kb)

Supplementary file8 The raw data of Fig. S2C. (XLSX 11 kb)

## Data Availability

All the data corresponding to the DLBCL datasets used in this study are available in GEO, GEPIA, and TCGA database. All of the experiment data in this study are available with reasonable requested.
