# Supplementary figures and images for: CCDC50, an essential driver involved in tumorigenesis, is a potential severity marker of diffuse large B cell lymphoma
Source: Ann Hematol. 2023 Sep 9;102(11):3153–65. doi: 10.1007/s00277-023-05409-w (PMC10567943; doi:10.1007/s00277-023-05409-w)

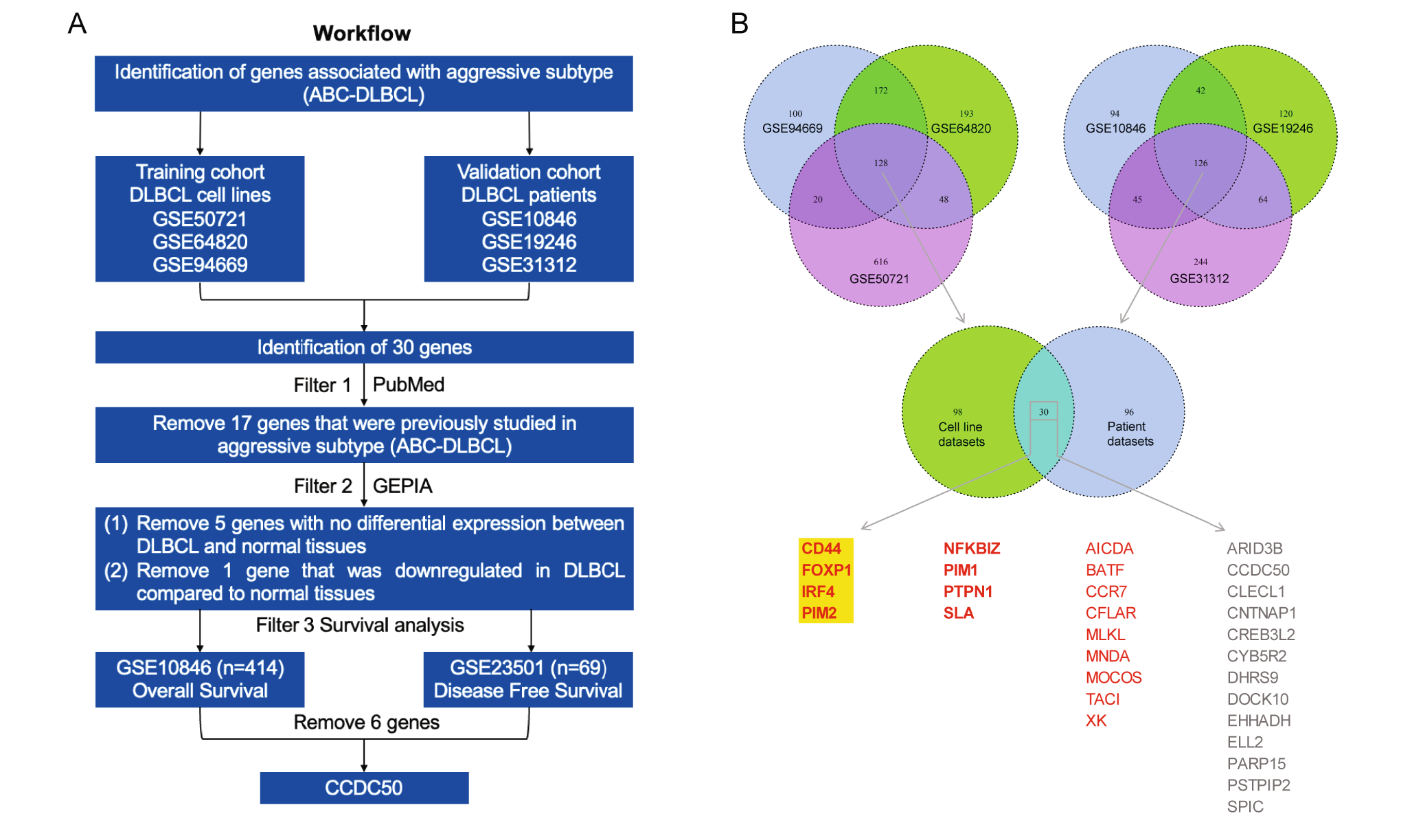

Supplement: Supplementary file 1 — Fig. S1 The screening process of ABC-DLBCL-associated genes. (A) The work flow of the screening process. (B)The four genes in the left column were well investigated in ABC-DLBCL, the four genes in the middle-left column were reported in ABC-DLBCL, the nine genes in the middle-right column were mentioned in ABC-DLBCL, the thirteen genes in the right column never be reported in ABC-DLBCL. (PNG 482 kb) [file 277_2023_5409_Fig7_ESM.png]

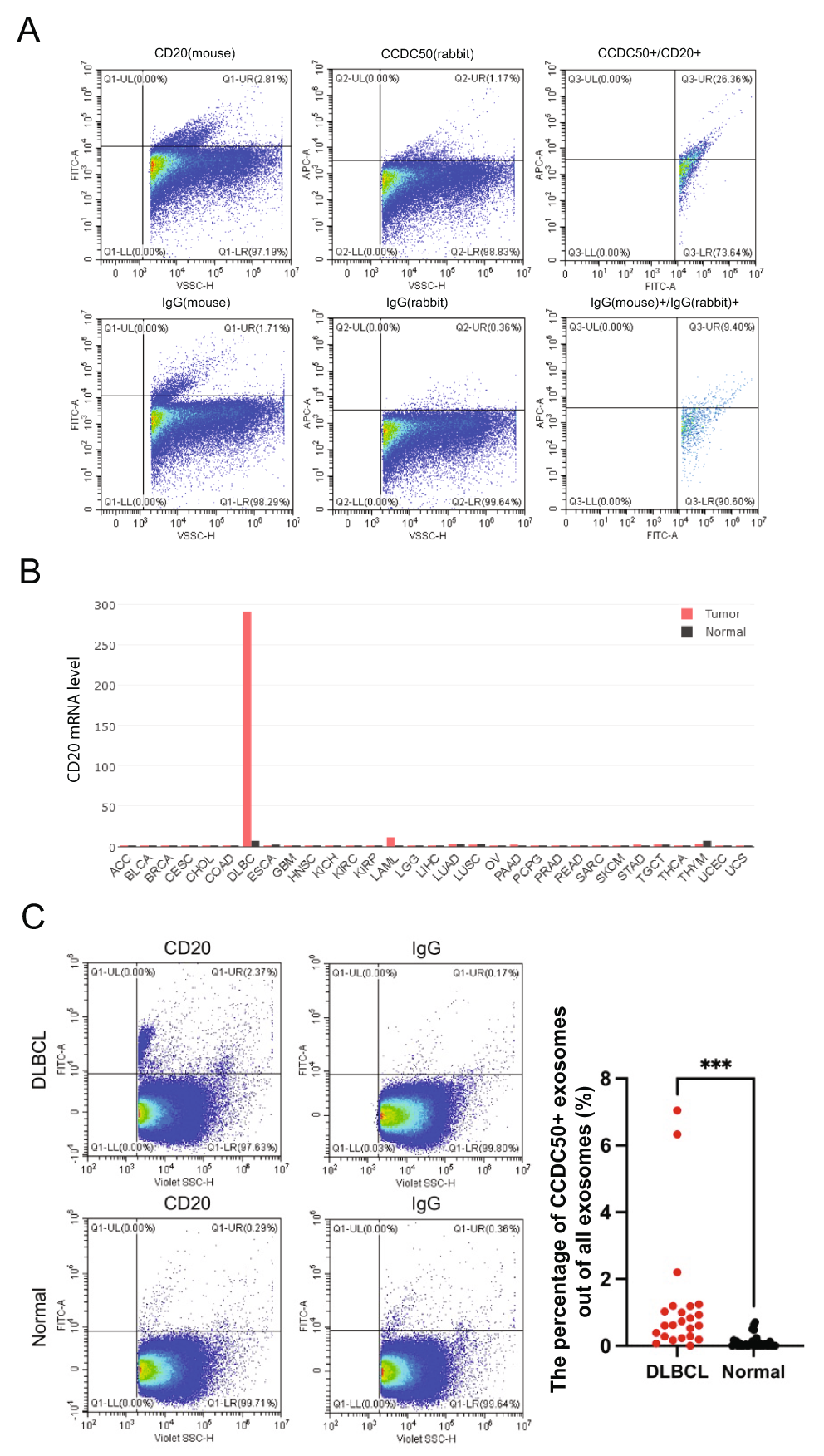

Supplement: Supplementary file 3 — Fig. S2 Detection of Exosomal CD20 and CCDC50 proteins. (A) Levels of exosomal CD20 and CCDC50 proteins and their corresponding controls (IgG) were detected in DLBCL patient samples by flow cytometry. (B) The expression level of CD20 in 31 types of cancer. (C) The percentage of CD20+ exosomes out of all exosomes were detected in DLBCL patients and normal samples. (PNG 725 kb) [file 277_2023_5409_Fig8_ESM.png]

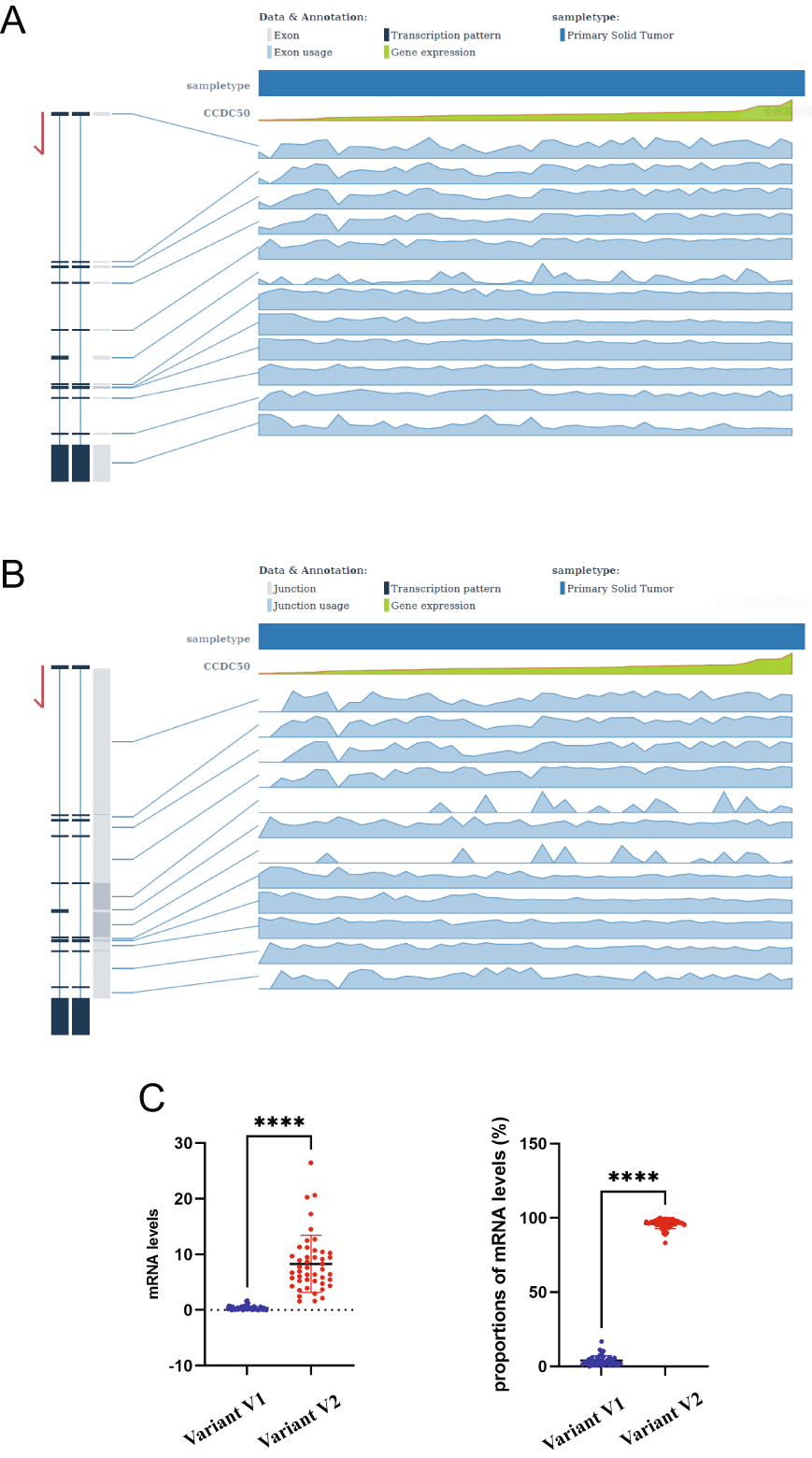

Supplement: Supplementary file 5 — Fig. S3 The expression level of CCDC50 V1 and V2 in DLBCL (TCGA). The expression level of each exon (A) and junction (B) of CCDC50 in DLBCL patients. (C) The expression level of CCDC50 variants and the proportion of each variant in DLBCL patients. (PNG 219 kb) [file 277_2023_5409_Fig9_ESM.png]
