# Supplementary material for: CCDC50, an essential driver involved in tumorigenesis, is a potential severity marker of diffuse large B cell lymphoma
Source: Ann Hematol. 2023 Sep 9;102(11):3153–65. doi: 10.1007/s00277-023-05409-w (PMC10567943; doi:10.1007/s00277-023-05409-w)
Supplement: Supplementary file 7 — Supplementary file1 (DOC 31 kb) [file 277_2023_5409_MOESM4_ESM.doc]

**Table S1** Sequence information of shRNA and primers

| **shRNA sequence** | |
| --- | --- |
| shCCDC50 (f) | GATCCGATGGAGGAATGAAGCCAAGACTCGAGTCTTGGCTTCATTCCTCCATCTTTTTTG |
| shCCDC50 (r) | AATTCAAAAAAGATGGAGGAATGAAGCCAAGACTCGAGTCTTGGCTTCATTCCTCCATCG |
| **Primers** | |
| c-Myc (f) | CACCGAGTCGTAGTCGAGGT |
| c-Myc (r) | TTTCGGGTAGTGGAAAACCA |
| CCDC50 (f) | GCTGGCTATTGAGGCAGAG |
| CCDC50 (r) | TGGCTTCATTCCTCCATCTT |
