# Supplementary material for: CCDC50, an essential driver involved in tumorigenesis, is a potential severity marker of diffuse large B cell lymphoma
Source: Ann Hematol. 2023 Sep 9;102(11):3153–65. doi: 10.1007/s00277-023-05409-w (PMC10567943; doi:10.1007/s00277-023-05409-w)
Supplement: Supplementary file 8 — Supplementary file2 (DOC 41 kb) [file 277_2023_5409_MOESM5_ESM.doc]

**Table S2** The information of antibodies.

| **Primary antibodies used in this study** | |
| --- | --- |
| CCDC50 | Abcam, ab127169 |
| CCDC50 | Santa Cruz, sc-398994 |
| beta-Actin | Santa Cruz, sc-47778 |
| c-Myc | Abcam, ab32072 |
| c-Myc (phospho S62) | Abcam, ab185656 |
| c-Myc (phospho T58) | Abcam, ab185655 |
| K48-Ub | CST, #4289 |
| GSK-3β | Abcam, ab32391 |
| GSK-3β (phospho S9) | Abcam, ab75814 |
| GSK-3β (phospho Y216) | NOVUS, NB100-81946 |
| p-PI3K (T458) | CST, #4228 |
| PI3K | CST, #4257 |
| Akt (pan) | CST, #4691 |
| p-Akt1 (S473) | Abcam, ab81283 |
| p-Akt1 (T308) | Abcam, ab278565 |
| FITC anti-human CD20 | Biolegend, 302304 |
| FITC Mouse IgG2b kappa Isotype Control | Invitrogen, 2204963 |
| **Secondary antibodies used in this study** | |
| Goat Anti-Mouse IgG (Dylight 800) | Abbkine, A23910 |
| Goat Anti-Rabbit IgG (Dylight 680) | Abbkine, A23720 |
| Mouse Anti-Rabbit IgG LCS | Abbkine, A25022 |
