# Supplementary material for: CCDC50, an essential driver involved in tumorigenesis, is a potential severity marker of diffuse large B cell lymphoma
Source: Ann Hematol. 2023 Sep 9;102(11):3153–65. doi: 10.1007/s00277-023-05409-w (PMC10567943; doi:10.1007/s00277-023-05409-w)
Supplement: Supplementary file 9 — Supplementary file3 (DOC 43 kb) [file 277_2023_5409_MOESM6_ESM.doc]

**Table S3** The information of clinicopathologic features of DLBCL patients

| **Category** | **Group** | **Number** |
| --- | --- | --- |
| **Gender** | Male | 224 |
|  | Femal | 172 |
| **Age(years)** | ≤60 | 188 |
|  | >60 | 226 |
| **Subtype** | GCB | 183 |
|  | ABC | 167 |
|  | Unclassified | 64 |
| **ECOG performance status** | 0 | 85 |
|  | 1 | 211 |
|  | 2 | 60 |
|  | 3 | 28 |
|  | 4 | 5 |
| **Stage** | 1 | 66 |
|  | 2 | 122 |
|  | 3 | 97 |
|  | 4 | 121 |
| **LDH ratio** | ≤ median ration(1.01) | 178 |
|  | > median ration(1.01) | 173 |
| **Number of extranodal sites** | 0 | 238 |
|  | 1 | 115 |
|  | 2 | 19 |
|  | 3 | 8 |
|  | 4 | 2 |
|  | 5 | 1 |

Abbreviations: GCB, germinal center B cell-like; ABC, activated B cell-like; ECOG: Eastern Cooperative Oncology Group.
