# Supplementary material for: CCDC50, an essential driver involved in tumorigenesis, is a potential severity marker of diffuse large B cell lymphoma
Source: Ann Hematol. 2023 Sep 9;102(11):3153–65. doi: 10.1007/s00277-023-05409-w (PMC10567943; doi:10.1007/s00277-023-05409-w)
Supplement: Supplementary file 10 — Supplementary file4 The process of exosome extraction and analysis. (DOCX 260 kb) [file 277_2023_5409_MOESM7_ESM.docx]

**(1) Exosome collection**

First, collect peripheral blood from DLBCL patients using EDTA anticoagulant tubes. Then, centrifuge at 800g for 5 minutes under 4°C and collect the upper layer of light-yellow plasma in a test tube. Continue to centrifuge at 3200g for 15 minutes under 4°C, collect the supernatant, which is the plasma, and remove any sediment. Divide the excess plasma into aliquots of 120uL each and store them at -80°C. Take 120uL plasma and place it in a 1.5mL centrifuge tube, centrifuge at 12000g for 30 minutes under 4 °C. Collect the upper layer of 100uL plasma and transfer it to a 1.5mL ultracentrifuge tube (BECKMAN, 357448), add 900uL PBS, mix well, centrifuge at 100000g for 70 minutes under 4 °C. Discard the upper layer of 800uL liquid, use a pipette to blow and mix the left 200uL liquid 200 times, then add 800uL PBS, mix well, centrifuge at 100000g for 70 minutes under 4 °C. Discard the upper layer of 800uL liquid, leaving 200uL liquid at the bottom of the centrifuge tube. Use a pipette to blow and mix the liquid 200 times to obtain a mixture rich in plasma-derived exosomes. Then we used Cryo-electron microscopy (Talos 120kV) and NTA scatter (Nanosight NS300) to determine whether we successfully isolated plasma exosomes from DLBCL patients.


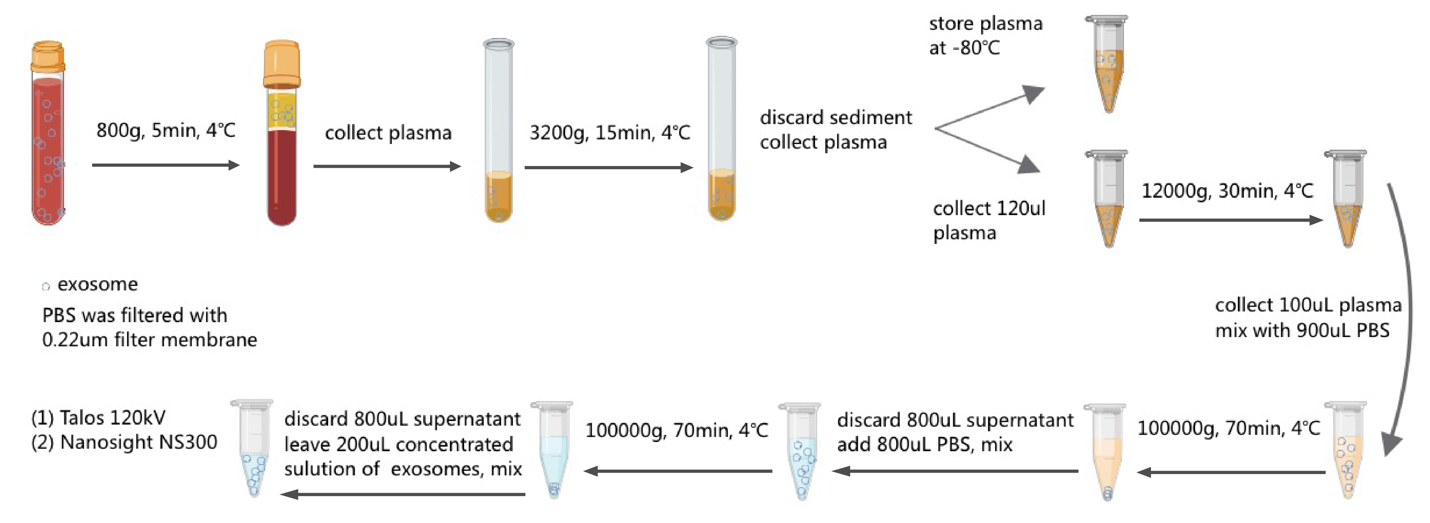


**(2) Exosome analysis**

Fluorophore-conjugated antibodies were generated using Zenon IgG labeling kits (Invitrogen) according to the manufacturer's protocol. Specifically, rabbit anti-CCDC50 monoclonal antibodies were labeled with Zenon Alexa Fluor 647. Rabbit anti-IgG monoclonal antibodies were also labeled as negative controls.

Labeled anti-CCDC50 (Alexa Fluor 647 labeled), together with FITC anti-human CD20 (Biolegend, 302304), were added to 10 μL exosome samples (equivalent antibody amount per sample: 0.06 μg) and incubated overnight at 4°C. Labeled sample was then fixed with 20 μL of 0.22 μm-filtered 4% paraformaldehyde for 20 minutes at room temperature, followed by dilution with 150 μL-filtered PBS. Finally, samples were analyzed using BECKMAN CytoFLEX (VSSC).
